# Toxicological Studies of Czech Beers and Their Constituents

**DOI:** 10.3390/foods8080328

**Published:** 2019-08-08

**Authors:** Tania Merinas-Amo, Rocío Merinas-Amo, Victoria García-Zorrilla, Alejandro Velasco-Ruiz, Ladislav Chladek, Vladimir Plachy, Mercedes del Río-Celestino, Rafael Font, Ladislav Kokoska, Ángeles Alonso-Moraga

**Affiliations:** 1 Department of Genetics, University of Córdoba, 14071 Córdoba, Spain; 2 Research and Teaching Brewery, Department of Technological Equipment of Buildings, Faculty of Engineering, Czech University of Life Sciences Prague, Kamýcká 129, 16500 Pargue, Czech Republic; 3 Department of Microbiology, Nutrition and Dietetics, Faculty of Agrobiology, Food and Natural Resources, Czech University of Life Sciences Prague, Kamýcká 129, 16500 Pargue, Czech Republic; 4 Agri-Food Laboratory, CAGPDS, Avda. Menéndez Pidal s/n, 14080, Córdoba, Spain; 5 Department of Crop Sciences and Agroforestry, Faculty of Tropical AgriSciences, Czech University of Life Sciences Prague, Kamýcká 129, 16500 Pargue, Czech Republic

**Keywords:** Czech beer, brewing, raw material, *Drosophila melanogaster*, HL-60 cell line, toxicity, antitoxicity, longevity, cytotoxicity, DNA damage

## Abstract

Background: Czech beers are unique because they are brewed using specific technology at a particular latitude and for being entirely produced in the area of the Czech Republic. The purpose of this work is the evaluation of toxicological effects of a variety of freeze-dried Czech beers, their raw materials (malts, hops and yeast) and processed-beer (wort, hopped wort and young beer). Methods: In vivo assays to evaluate the safety and protective effects in the *Drosophila melanogaster* eukaryotic system, and the in vitro evaluations of chemopreventive and DNA damage activity using the HL-60 tumour human cell line were carried out. Results: The safe effects for all the analysed substances and general protective effects against H_2_O_2_ were shown both at the individual and genomic level in the *Drosophila* animal model, with some exceptions. Moreover, all the substances were able to inhibit the tumour cell growth and to induce DNA damage in the HL-60 cells at different levels (proapoptotic, single/double strands breaks and methylation status). Conclusions: The promising effects shown by freeze-dried Czech beers due to their safety, protection against a toxin, chemopreventive potential and the induction of DNA damage in tumour cells, allow the proposition of Czech beer as a beverage with nutraceutic potential.

## 1. Introduction

Beer is one of the oldest known beverages. Approximately 6000 years ago, ancient texts revealed the first beer production by the Sumerians: “the sweetest grain, if baked, left out, moistened, forgotten, and then eaten, would produce an uplifting, cheerful feeling” [1]. The different contributions like the use of hops, yeast, the different fermentation methods and the high technology development in the last century have contributed to the growth, improvement and variety of brewing as is currently known [1,2,3].

Beer is not only considered as a beverage, but it provides a valuable added value to the diet. It acts as a nutritional supplement with many bioactive molecules, as it contains readily available starches and sugars, various minerals and valuable B vitamins [2]. The brewing industry is a global business in traditional European brewing regions with local varieties of beer still maintained mostly in central Europe [4].

The beer industry of the Czech Republic (Bohemia) has a long history since the first Czech brewery was founded in 993 at Břevnov Monastery in Prague [5], and it was only in October 2008, when the EU allowed the Czech Republic to use the “Ceské pivo/Czech Beer” trademark [6].

Czech beer is unique because it is brewed using specific technology and at a particular latitude. Due to the specific character of Czech beer, that is, the water, malt, hops and yeast used in the brewing, Czech brewers want recognition of the uniqueness of their beer.

Many studies highlight the value and potential uses of beer and its components. Some of the beers are those derived from raw materials (water, malts, hops and yeast) and pass unchanged through the brewing process, and some others are produced during the process [7]. Chen et al. [8] emphasised the potential uses of beer compounds in dermatology, such as in the treatment of atopic eczema, contact dermatitis, pigmentary disorders, skin infections, skin ageing, skin cancers and photoprotections. Sánchez et al. [9] highlighted the common use of hops as a tranquillizer plant. Further, it is known that both the normalized and stimulated effects of the many compounds of beer have on the sleep’s rhythm because of its bitter acids, especially the alpha acid components [8]. Furthermore, others studies support the benefits of moderate alcohol consumption, being that a healthy lifestyle is associated with lower rates of cardiovascular diseases [10], or the ability against kidney stone formation [11]. Moreover, Vinson et al. [12] reported other activities of lager and dark beers, like the inhibition of atherosclerosis, lowering both the total serum cholesterol and triglycerides, to act as in vivo antioxidants by decreasing the oxidizability of low-density lipoprotein cholesterol and decreasing atherosclerosis in cholesterol-fed hamsters. These activities are mainly due to the polyphenols present in both lager and dark beer. Nevertheless, the adverse effects of alcohol on health, such as increasing risk of several mouth, oesophageal and liver cancers [13], should be noted.

Despite beers being considered beverages with important properties on health [8,12,14,15,16,17], nowadays studies about the toxicological evaluation of beers are missing. Moreover, due to the traditional history of beer in the Czech Republic, the present work attempts to add new data about the toxicological properties of a wide variety of Czech beers, as well as their unique and differential raw materials (malts, hops and yeast) and further, processed-beer at three different steps during its brewing (wort, hopped wort and young beer). For that purpose, a previous assay of the safety of freeze-dried samples followed by studies on the protective effects that the tested compounds show in the *Drosophila* animal model were carried out. Nonetheless, chemopreventive studies in the HL-60 human cell line were carried out to evaluate the possible cytotoxic potential and DNA damage that our tested compounds could induce in tumour cells.

− The fruit fly *D. melanogaster* shows a consistent similarity to humans as approximately 75% of the genes involved in diseases are similar in the fly [18,19]. This well-known eukaryote with the largest scientific history in genetics has also contributed to the understanding of developmental biology, evolutionary concepts and more recently, toxicology [20,21,22,23,24]. Its short life cycle (10–12 days), cost efficiency and easy maintenance are characteristics that make *Drosophila* to be considered as an ideal model organism.
− The HL-60 human model cell line originated thirty two years ago from a patient with acute myeloid leukaemia [25]. After culturing peripheral blood leukocytes from this female patient, a growth-factor-independent immortal cell line with distinct myeloid characteristics was obtained [26]. This promyelocytic leukaemia cell line has been used world-wide for many toxicity and cancer purposes.

## 2. Materials and Methods

### 2.1. Samples Preparation and Sample Compounds

For the present study, 16 samples of lager type beers commercially produced in Czech Republic were purchased at a local market (see Table 1). Moreover, 6 raw materials and 3 processed-beer samples were obtained from the Research and Teaching Brewery of the Czech University of Life Sciences, Prague. This consists of a two-roll mill, brew house (steam heated kettle 12 hl, and lauter tun 14 hl), whirlpool 12 hl, wort cooler (10 hl/h), 6 fermentation tanks (24 hl each), and 14 lager tanks (22 hl). The standard brewing conditions were as follows: 200 kg Pilsen malt, 2 mash process, 3 kg of Saazer hop variety, fermentation at 12 °C for 7 days, yeast W96, maturation at 0 °C for 3 months. The annually brewery production is 300–500 hl beer. Although Suchdol Jenik is not a commercial brewery, it is built on the tradition of beer brewing in Prague’s Suchdol using the common raw materials farmed in the Czech Republic. A total of 400 mL of every sample of beer was used to be freeze-dried. The detailed characteristics of all 25 different samples analysed in this study are summarised in Table 1. The beer samples were lyophilised at −110 °C until the samples were totally dried using a Scanvac CoolSafe 110-4 freeze-dried (LaboGene ApS, Lynge, Denmark) before carrying out the assays and stored in dark and dried conditions until use. The lyophilised samples were stored in a sterilised plastic bottle with the correspondent information (name, date and quantity of beer). All lyophilised beers were dissolved and distilled.

The concentrations of the tested beers were established according to the average daily intake and body weight of *Drosophila* [27]. The concentration range was stablished to make it equivalent to the 192 mL of total beer/day in humans [28].

### 2.2. In Vivo Animal Model and In Vitro Cellular Culture Used

#### 2.2.1. Drosophila Melanogaster

Two *Drosophila* genetic backgrounds were used in our studies:

− *mwh/mwh*: Carries a recessive mutation *mwh* (*multiple wing hairs*) which produces several tricomas instead of one per cell when it is in homozygosis [30].
− *flr^3^/In (3LR) TM3, rip^p^sep bx^34e^e^s^Bd^S^*: Carries a *flr^3^* (*flare*) marker which is a recessive lethal mutation when it is in homozygosis producing deformed tricomas, though it is viable in homozygous somatic cells if and when the larvae start the development [31].

The strains were routinely maintained in the laboratory at 25 °C in a homemade meal, and serial changes were made.

The direct and reciprocal crosses between both strains of *Drosophila* were stablished and the use of the emerging trans-heterozygous adults (*mwh flr^+^/mwh^+^ flr^3^*) were used for the in vivo toxicity, antitoxicity, genotoxicity, antigenotoxicity and lifespan assays.

#### 2.2.2. HL-60 Cell Cultures

The cells were grown in a conditioned RPMI-1640 medium (Sigma, Darmstadt, Germany, R5886) which was complemented with decomplemented foetal bovine serum (Linus, S01805), L-glutamine (Sigma, G7513) and an antibiotic-antimycotic solution (Sigma, A5955). Routinely, the cells were grown in a 37 °C and 5% CO_2_ [26] in a Shell Lab CO_2_ incubator. The cultures were seeded at 2.5 × 10^4^ cells/mL density and renewed three times per week.

### 2.3. Safety In Vivo Assays of Freeze-Dried Samples

#### 2.3.1. Toxicity

The number of emerged adults in each treatment with respect to the negative control was analysed at the following ranks of concentrations for the different lyophilised beers: 3.125–50 mg/mL. For the malts, hops and yeast, the concentrations used were those equivalent to the quantity of each compound in the full beer (0.0625–1 mg/mL for blond and dark malts; 0.00156–0.025 mg/mL for Sladek hop; 0.000625–0.01 mg/mL for Saazer hop; 0.00023–0.00375 mg/mL for *S. uvarum*; 3.125–50 mg/mL for processed-beers). The tester tubes contained *Drosophila* Instant Medium (DIM) (Formula 4–24, Carolina Biological Supply, Burlington, NC, USA) and 4 mL of the different assayed concentrations. Moreover, the negative concurrent controls were prepared with DIM and distilled water.

The toxicity significances were assessed with the non-parametric Chi-square test [32].

#### 2.3.2. Genotoxicity

Due to the similar pattern observed for the different beers studied in the toxicity assay, a blond beer was selected to study it effects on the *Drosophila* genome. It was chosen according to today’s most known and oldest Czech breweries: Pilsner Urquell (1842) [33]. Moreover, the studies with raw materials and processed-beer at different concentrations were carried out in this animal model.

For the genotoxicity assays, the method described by Graf et al. [34] were applied. As in the toxicity test, the assayed tubes contained DIM and the solutions of the substances to be tested (3.125 and 50 mg/mL of lyophilised beer; 0.0625 and 1 mg/mL for blond and dark malts; 0.00156 and 0.025 mg/mL for Sladek hop; 0.000625 and 0.01 mg/mL for Saazer hop; 0.00023 and 0.00375 mg/mL for *S. uvarum*; 3.125 and 50 mg/mL for processed-beers). A positive control, consisting of DIM, water and 0.12 M H_2_O_2_ as a genotoxicant, was tested to check the accuracy of the assay. After emergence, the adult flies were preserved in a solution of 70% ethanol until the next wing step.

The mutations were scored at 400× in the trans-heterozygous mounted wings. Given the substantial knowledge about the origin of the mutations, the total mutations were spliced into different categories according to their size and the type of mutation: *mwh* phenotype with small single spots (1–2 cells) that occur in the late stages of the mitotic division; *mwh* or *flr^3^* phenotype with large single spots (≥3 cells) that occur in the early mitotic divisions of larval development; and both *mwh* and *flr^3^* twin juxtaposing spots. On the one hand, small and large spots are induced by somatic point mutations, chromosome aberrations, and somatic recombinations, whereas it is assumed that twin spots appear only by somatic recombinations between the centromere and the *flr^3^* locus.

A total of 736 wings were mounted and analysed for the genotoxicity treatments. The number of mutations were compared to the water control and a double decision test was applied [35], and the binomial Kastenbaum and Bowman statistic test were applied [36]. The non-parametric Mann Whitney *U*-test (*α* = *β* = 0.05) was used to solve the inconclusive and positive results.

### 2.4. Protection In Vivo Tests

#### 2.4.1. Antitoxicity

The antitoxicity tests in the study consisted of the viability percentage of *Drosophila* treated with the same concentrations as in the above described toxicity assays but combined with 0.12 M H_2_O_2_ as a toxicant [37].

The statistical significances assessing the effects of the tested compounds on the survival of flies were given by the Chi-square test in Microsoft Office Excel 2007 [32].

#### 2.4.2. Antigenotoxicity

The antigenotoxicity trials were carried out as Graf et al. [38] described, and consisted of the combined treatments with 0.12 M H_2_O_2_ as a genotoxin and the tested compounds (using the same conditions as in the genotoxicity assay). After the emergence of the adult flies, the same protocol of genotoxicity was followed for the mounted and scored wings. A total of 741 wings were mounted and analysed. The inhibition percentages of the mutagenic activity (IP) in the antigenotoxicity trials were obtained from the total spots per wing following the Abraham (1994) algorithm [39]:

IP = [(genotoxin − combined treatment)/genotoxin] × 100

#### 2.4.3. Longevity

Based on the similar pattern observed for the different beers studied in the toxicity and antitoxicity assays, a blond and a stout beer was selected to study their effects on the *Drosophila* life extension. It was chosen according to today’s most known and oldest Czech breweries: Pilsner Urquell (1842) [33]. Moreover, the studies with raw materials and processed-beer at different concentrations were carried out in the model organism.

All longevity assays were carried out at a constant temperature (25 °C) following the protocol described by Tasset-Cuevas et al. [40]. Briefly, the groups of 25 individuals were collected and placed into vials that contain 0.21 g of DIM and 1 mL of the different concentrations to be tested for each compound (3.125–50 mg/mL of lyophilised beer; 0.0625–1 mg/mL for blond and dark malts; 0.00156–0.025 mg/mL for Sladek hop; 0.000625–0.01 mg/mL for Saazer hop; 0.00023–0.00375 mg/mL for *S. uvarum*; 3.125–50 mg/mL for processed-beers). Four replicas were carried out during the longevity assay for each control and the concentration established, recording the number of alive animals and renewing the culture media every 3/4 days.

In addition, the quality of life of treated flies was known by studying the upper 25% of the survival curve. This part of the longevity curves is considered as the healthspan, defined as a low and more or less constant age-specific mortality rate value [41].

The statistical analysis of the survival data was carried out by applying the Kaplan-Meier methodology (SPSS Statistics 17.0 software, SPSS, Inc., Chicago, IL, USA). The Log-Rank (Mantel-Cox) post hoc method was used to solve the differences among the survival curves.

### 2.5. Chemopreventive In Vitro Tests

#### 2.5.1. Cytotoxicity Assay

The HL-60 cells at 2 × 10^4^ cells/mL density were placed in 96 well culture plates and treated for 72 h with the different lyophilised beers (1.25–250 mg/mL) and the equivalent concentrations for the raw materials (0.156–20 mg/mL for both malts; 0.00097–0.125 mg/mL for Sladek hop; 0.00039–0.05 mg/mL for Saazer hop; 0.00029–0.0375 mg/mL for *S. uvarum*) and the processed-beers (1.25–250 mg/mL). This wide range was selected to evaluate the in vitro cytotoxic doses using the inhibitory concentration of 50 (IC_50_) when possible.

The trypan blue dye exclusion test was used to determine the cell viability. Trypan blue (Sigma-Aldrich, St. Louis, MO, USA, T8154) was added to the cells at a 1:1 volume ratio, loaded into a Neubauer chamber, counted at 100× magnification (AE30/31, Motic microscope, Cabrera de Mar Barcelona, Spain). Finally, the curves were represented graphically as a survival percentage from three independent experiments with respect to their concurrent control.

#### 2.5.2. Tumour Cells DNA Damage Evaluation

Due to the similar pattern observed for the different beers studied in the in vivo assays, a blond beer was selected to study the DNA damage evaluation. Pilsner Urquell (1842) was chosen according to today’s most known and oldest Czech breweries [33]. Moreover, the studies with raw materials and processed-beer at different concentrations were carried out in human leukaemia cells.

##### DNA Fragmentation

The treated HL-60 cells (1 × 10^6^/mL) during 5 h with blond selected lyophilised beer (1.25–250 mg/mL) and its correspondent concentration for the raw materials (0.156–20 mg/mL for both malts; 0.00097–0.125 mg/mL for Sladek hop; 0.00039–0.05 mg/mL for Saazer hop; 0.00029–0.0375 mg/mL for *S. uvarum*) and the processed-beers (1.25–250 mg/mL) were centrifuged (3000 rpm for 5 min). DNA was extracted according to the protocol described by Merinas-Amo et al. [42].

For the quantification of DNA, a spectrophotometer (Nanodrop ND-1000) was used. Finally, the quantity of 1200 ng of DNA was subjected to an agarose gel (2%) electrophoresis at conditions of 85 mA for 25 min, stained with GelRed^TM^ and visualized under UV light.

##### Comet Assay

The cells were incubated for 5 h with different concentrations of the selected lyophilised beer (7.56, 31.25 and 62.5 mg/mL) and its equivalent concentrations for the raw materials (0.625, 2.5 and 5 mg/mL for both malts; 0.0039, 0.0156 and 0.03125 mg/mL for Sladek hop; 0.00156, 0.00625 and 0.0125 mg/mL for Saazer hop; 0.00117, 0.0047 and 0.0094 mg/mL for *S. uvarum*) and the processed-beers (7.56, 31.25 and 62.5 mg/mL). As in the DNA fragmentation assay, two representative blond (Pilsner Urquell) and stout (Staropramen Cerny) Czech beers were selected for this study.

After completing the washing steps in PBS (Phosphated-Buffered Saline), 6.25 × 10^5^ cells/mL were mixed with low melting point agarose (Sigma, A4018) at 0.75% and immediately transferred to frosted-end slides. Next, according to the Mateo-Fernández et al. [43] protocol, the steps of lysis, alkaline electrophoresis, neutralization and drying were carried out. Finally, 50–100 single cells were visualized by treating the slides with 7 µL of propidium iodide (10 µg/mL) (Sigma, P4170). The DNA comet images were analysed (400×) in a Leica DM 2500 fluorescence microscope with a green filter and an attached camera (JAI CV-M4CL, Barcelona, Spain). The single cell parameters were calculated using the OpenComet plugging from ImageJ (NIH).

The statistical analysis of the tail moment was evaluated by an ANOVA, and a post hoc Tukey’s test was applied to assess the effect that the different compounds had on the DNA integrity. Significance was considered at *p* ≤ 0.05 (SPSS Statistic 17.0 software).

##### Methylation Status Evaluation

For the evaluation of the methylation status of the treated tumour cells, firstly the genomic DNA was isolated in the same way as described in the DNA fragmentation section. Secondly, a bisulphite-modified DNA step from the different treatments selected for the lyophilised beer (7.81 and 125 mg/mL), raw materials (0.625 and 10 mg/mL for blond and dark malts; 0.0039 and 0.0625 mg/mL for Sladek hop; 0.00156 and 0.025 mg/mL for Saazer hop; 0.00117 and 0.0188 mg/mL for *S. uvarum*) and processed-beer (7.81 and 125 mg/mL) was carried out using the EZ DNA Methylation-Gold Kit. Thirdly, a quantitative Methylation-Specific PCR (qMSP) was carried out following the methodology described by Merinas-Amo et al. [44] in a MiniOpticon Real-Time PCR System (MJ Mini Personal Thermal Cycler, Bio-Rad Laboratories Inc., Hercules, CA, USA) and analysed by the Bio-Rad CFX Manager 3.1 Software. The reaction mixture consisted of: 2 µL of deionised water, 5 µM of each forward and reverse primer, 2 µl of iTaq™ Universal SYBR^®^ Green Supermix (Bio-Rad, containing antibody-mediated hot-start iTaq DNA polymerase, dNTPs, MgCl_2_, SYBR^®^ Green I dye, enhancers, stabilizers and a blend of passive reference dyes including ROX and fluorescein) and 25 ng of bisulphite converted genomic DNA up to a 10 µL final volume and submitted to specific qMSP conditions (95 °C for 3 min, 45 cycles at 95 °C for 10 s, 60 °C for 15 s, 72 °C for 15 s, a step at 95 °C for 30 s following by a 65 °C step during 30 s and finally a boost step from 65 °C to 95 °C for 95 s increasing 0.5 °C each 0.05 s).

A wide range of human genomic DNA could be evaluated across repetitive sequences. Alu and LINE sequences belong to the interspersed repeat sequences throughout the genome, while the satellites are located in the centromere areas [45,46,47,48]. The Alu M1, LINE-1 and Sat-α sequences were obtained from Isogen Life Science (see Table 2 for further information [49]).

The relative expressions obtained from three replicas of each sample were normalised with the Alu C4 housekeeping sequence using the Nikolaidis et al. [50] and Liloglou et al. [51] comparative C_T_ method. To evaluate the statistical differences between the control and each treated group, the one-way ANOVA and post hoc Tukey’s tests were used.

## 3. Results and Discussion

### 3.1. Safety of Freeze-Dried Samples

**Figure 1:**
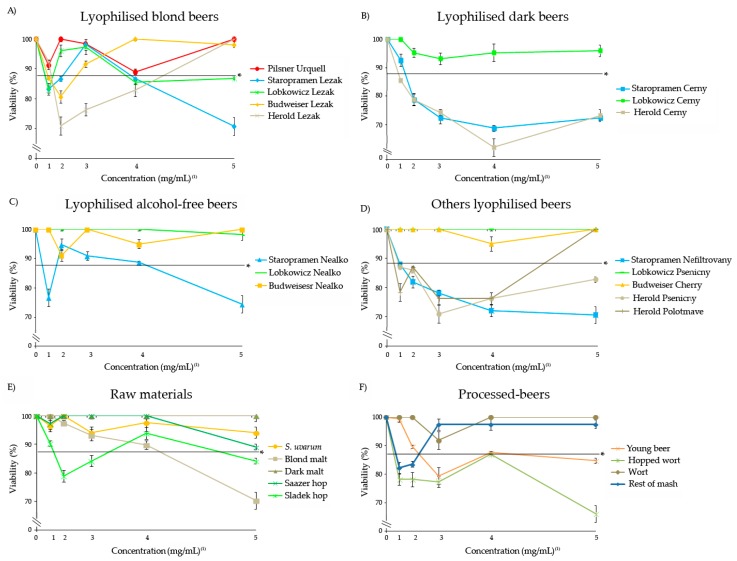
The toxicity levels of lyophilised Czech beers (**A–D**), raw materials (**E**) and processed-beers (**F**) in *D. melanogaster*. The data represent the percentage of surviving adults with respect to 300 control untreated 72-h-old larvae from three independent experiments ± SE treated with five concentrations of lyophilised Czech beers, raw materials and processed-beers. *: Chi-square value (*p* < 0.05). The values 1, 2, 3, 4 and 5 correspond to the concentrations of 3.125, 6.25, 12.5, 25 and 50 mg/mL for beers and their respective concentrations for each single component.

The toxicity of Czech lyophilised beers, raw materials and processed-beer has been carried out in the *Drosophila* in vivo model. The relative percentage of emerging flies after the treatments with different concentrations of the substances is shown in Figure 1. None of the substances reached the LD_50_ thus all of them can be considered non-toxic in the *Drosophila* animal model. Nevertheless, the viability rates were different.

The highest reduction of *Drosophila* viability caused by the tested beer samples and their constituents reached a 63.4% of survival, compared with the control. Taking into account that a toxic effect is considered when survival reaches a reduction of under 50% of survival, all our samples exhibited a non-toxic/safe effect on the animal model. On the other hand, if the survival of flies is analysed with a *p* < 0.05, the results showed different patterns between the control and the treatments: (i) The samples with a safety potential significantly similar to the control in all the concentrations (Pilsner Urquell; Lobkowicz Cerny; Lobkowicz Nealko; Lobkowicz Psenicny; Budweiser Nealko; Budweiser Cherry; Dark malt; Saazer hop; *S. uvarum* and Wort); (ii) the samples with a safety potential at the lowest/medium concentrations (Staropramen Cerny; Herold Lezak; Blond malt; Sladek hop and Young beer); (iii) the samples with safety properties at the highest/medium concentrations (Rest of mash; Herold Lezak; Herold Polotmave; Budweiser Lezak; Sladek hop); (iv) the samples with safety properties at medium concentrations (Staropramen Lezak; Staropramen Nealko; Lobkowicz Lezak) and (v) the samples with no safety properties at any concentrations with respect to the control (Staropramen Nefiltrovany; Herold Cerny; Herold Psenicny; Hopped wort).

To the best of the authors’ knowledge, this is the first study on the toxicity of Czech beers using *D. melanogaster*. However, some studies carried out with prokaryotes using the Ames test which tested other types of beers suggested the possibility that the antimutagenic activity showed by these drinks is a result of an accumulated effect of their individual constituents [52]. Moreover, phenolics and some B vitamins have been argued to be health promoter compounds of beer in reference to degenerative diseases [53]. Our results agree with alleged safety properties of freeze-dried beer and its components adding more information about the promising properties that specifically Czech beers show as nutraceutic substances in a model organism.

### 3.2. Genotoxicity of Freeze-Dried Samples

Table 3 shows the results of genotoxicity in the somatic mutation and recombination test (SMART) of *D. melanogaster*. The negative control showed a rate of 0.158 mutations/wing [54] which falls into the historical range for the wing spot test. All results showed non-genotoxic effects at the assayed concentration with a non-significant frequency of mutations per wing ranging between 0.125 and 0.368 with respect to the negative control. Moreover, a positive control to check the assay accuracy was carried out with a combined treatment prepared with DIM, water and 0.12 M H_2_O_2_ as an oxidative genotoxicant. As indicated in the antigenotoxicity section, the results fall into the established range as genotoxic substances [54].

No information has been found about genotoxicity related to raw materials and processed beers. Hops seems to be one the most genoprotective compounds among the assayed ones, and may be conferring beer its genoprotective properties. This fact is in agreement with other authors, who pointed that Xanthohumol, a main flavonoid found in hops, has a strong antimutagenic activity [36,55]. Furthermore, Arimoto-Kobayashi et al. [56] suggested that beer components act in a protective capacity against the genotoxic effects of heterocyclic amines in vivo. A previous study with a lyophilised blond lager beer on *Drosophila* suggested that the values of a total mutation frequency were similar or lower than the negative control with no significant differences between them [36].

### 3.3. Antitoxicity

**Figure 2:**
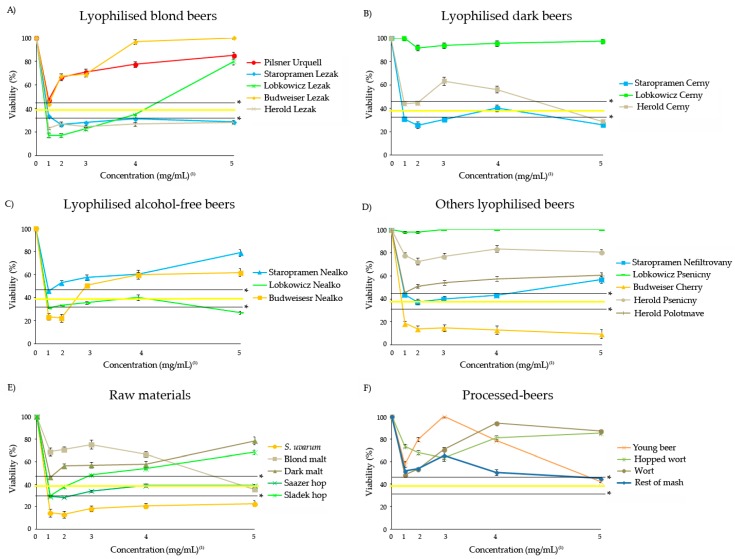
The antitoxicity levels of different lyophilised Czech beers (**A–D**), raw materials (**E**) and processed-beers (**F**) in *D. melanogaster*. The data represent the percentage of surviving adults with respect to 300 control untreated 72-hour-old larvae from three independent experiments ± SE treated with different concentrations of lyophilised Czech beers, raw materials and processed-beers combined with 0.12 M H_2_O_2_. *: Chi-square value (*p* < 0.05). The value 1, 2, 3, 4 and 5 correspond to concentrations of 3.125, 6.25, 12.5, 25 and 50 mg/mL for beers and their respective concentrations for each single component. The positive control of H_2_O_2_ 0.12 M is represented as yellow line showing a toxic effect with an average survival rate of 39.2% with respect to the negative control.

The antitoxicity assays revealed the ability of the Czech lyophilised beers, raw materials and processed-beers to protect the individuals against an oxidative stress at the tested concentrations. Figure 2 shows that the positive control H_2_O_2_ is toxic at 0.12 M, with respect to the negative water control, with an average survival percentage of 39.2%.

All compounds showed a protective effect with respect to their concurrent control except *S. uvarum*, Budweiser Cherry and Staropramen Nefiltrovany at all the tested concentrations. Moreover, no antitoxic activity against H_2_O_2_ was shown at lower concentrations of Sladek hop, Staropramen Nealko and Staropramen Cerny, and at higher concentrations of Herold Cerny, Lobkowicz Nealko and Staropramen Cerny. On the other side, a significant protective effect was shown at combined treatments with Pilsner Urquell, Lobkowicz Cerny, Lobkowicz Psenicny, Budweiser Lezak, Herold Lezak, Herold Psenicny, Herold Polotmave, rest of mash, blond malt, dark malt, wort, hopped wort and young beer; at lower concentrations of Staropramen Lezak; at medium/higher concentrations of Herold Cerny; and at higher concentrations of Staropramen Nealko, Lobkowicz Lezak, Sladek hop, Saazer hop, Budweiser Nealko.

Due to the ability of beers to increase antioxidant capacity in humans, many studies focused on the evaluation of their possible benefits, since oxidative stress and consequently the presence of reactive oxygen species are closely associated with diverse types of diseases [57]. To the authors’ knowledge, this is the first study on the antitoxicity of Czech beers and its constituents using *D. melanogaster*.

It is known that the antioxidant capacity of beer is mainly derived from polyphenols, Maillard compounds and vitamin C from malt and hops [17,58,59,60,61,62]. Nevertheless, it is also known that the Maillard reaction can cause both deterioration of food quality [63,64,65], by the formation of anti-nutritional and toxic products (MRPs) with mutagenic [66], carcinogenic [67] and cytotoxic [68] effects. Our results agree with this fact as both malts and hops showed protective effects against H_2_O_2_ with respect to their concurrent controls, except at the lowest concentration of Sladek hop tested.

The antioxidant power of beer depends on a wide variety of parameters involved in brewing, like the malt variety and the malting process, the mashing temperature and pH, sparing, boiling, the hops variety added during wort boiling and yeast fermentation [17]. According to Rivero et al. [69], dark beer has a higher content of antioxidants than lager or alcohol-free beer, which are responsible for its colour. Moreover, because of the brewing process, the antioxidant capacity of alcohol-free beer is considerably lower than lager [17]. No relationship has been found between the antioxidant activity of phenols and the degree of browning [70]. Our results did not establish any pattern about the characteristics of beers and their antioxidant potential: A stout, an unfiltered, an alcohol-free and a special cherry beer, together with *Saccharomyces* were the only four substances among a total of 26 samples studied which did not show protective effects against the hydrogen peroxide oxidative model toxin. This study hypothesised that antioxidant activities shown by beers are caused by the synergic activities of the different phenols that compose them. Previous results supported our result that no correlation was established by the different styles of beer studied in the in vivo assays. However, better and safe properties for lyophilized beer are shown by the blond ones, followed by alcohol-free and dark beers [71,72].

The antioxidants present in hops are also important for the brewing industry. A study confirms the different composition of both Saazer and Sladek hop resins [73]. Saazer fine aroma hops has a similar composition of hop resins owing to genetic affinity. The content of α-bitter acids ranged between 3–4.5% *w/w* and was lower than the content of β-bitter acids (4.5–6.5% *w/w*). Sladek hybrid aroma varieties hop resins have a composition of 4–8% *w/w* of α-bitter acids and 3.5–8% *w/w* of β-bitter acids. Moreover, different relative percentages of antioxidant activity has been observed in these hops which correlated with the content of polyphenol substances [74]. The highest antioxidant activity within 70 to 80% rel. was exhibited by the variety Saazer, followed at a relatively considerable distance by the Sladek variety with an antioxidant activity within 40 to 60% rel. Our results agree with this characterisation of hops, showing a stronger antitoxic potential for Saazer hop at the lowest concentrations tested.

### 3.4. Antigenotoxicity

The results of antigenotoxicity obtained in the SMART test are shown in Table 4. Oxygen peroxide (0.12 M) behaves as a potent mutagen able to induce somatic mutations in *D. melanogaster* [75] at a rate of 0.388 spots/wing.

The results showed that all compounds were able to inhibit the genotoxic activity of H_2_O_2_, with the exception of the highest concentration of Blond malt and Sladek hop, the lowest concentration of *S. uvarum* and Hopped wort, and both tested concentrations of Young beer that showed no-significant differences with respect to the positive control. An inhibition percentage that ranged between 16.75 and 75 was induced by the compounds corresponding to the lowest concentration of *S. uvarum* and the highest concentrations of Saazer hop, respectively. In addition, the most antigenotoxic potential against hydrogen peroxide was shown by Saazer hop followed by Dark malt, Wort, Pilsner Urquell, Hopped wort, Sladek hop, Blond malt, *S. uvarm* and finally, by Young beer as shown in Table 4.

To the authors’ knowledge, this is the first time that the antigenotoxic effects of raw materials and processed-beers in the in vivo tests have been studied. From the results obtained, beers and their constituents are not only not genotoxicants, but also protect from the damage to DNA caused by oxidative genotoxicants. A previous study with a lyophilised blond lager beer and some of its compounds indicated an anigenotoxic effect supporting our results [42].

Moreover, it is known that individual nutrients or other chemicals derived from food have effects on chronic diseases which may not show the same effects as the whole nutrient [76]. This may the reason why no-relationship could be stablished between the compounds in this study.

### 3.5. Longevity

**Figure 3:**
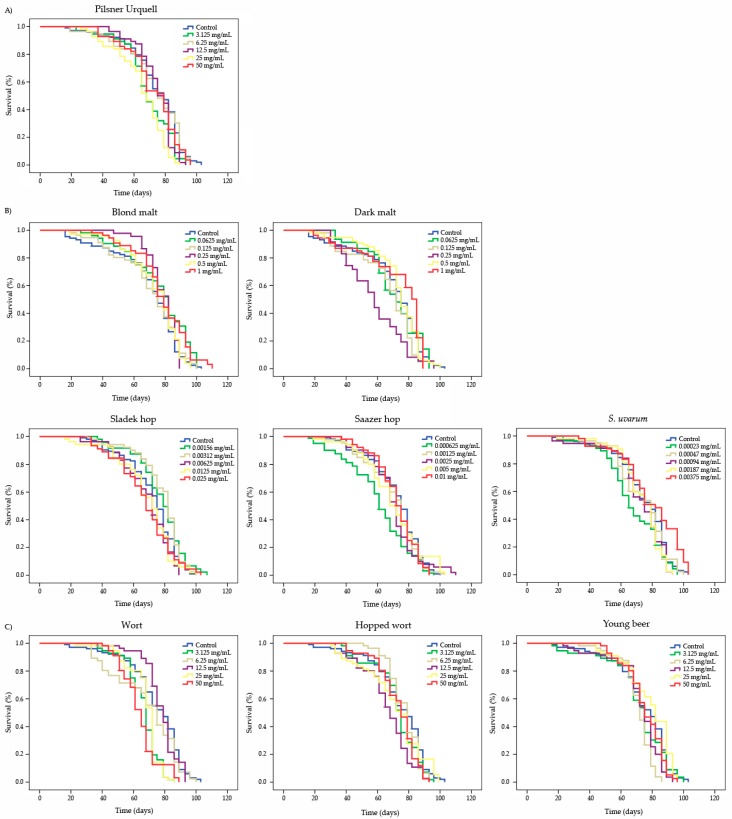
The survival parameters of *D. melanogaster* fed with different concentrations of a lyophilised Czech beer (**A**), raw materials (**B**) and processed-beers (**C**). The curves were obtained by the Kaplan-Meier method and the significances of the curves were determined by the Log-Rank method (Mantel-cox).

The longevity parameters assayed in the *D. melanogaster* model for the four compounds tested are shown in Figure 3 and Table 5. The survival curves showed a significant expansion of life at both extreme concentrations of blond malt, at two-lowest concentrations of Sladek hop and the highest concentration of *S. uvarum* tested. A significant reduction in lifespan was only induced by the lowest and fourth-to high concentrations of Pilsner Urquell, at second and third-to low concentrations of dark malt and young beer, at medium concentrations of Saazer hop and Hopped wort, at lowest concentrations of *S. uvarum* and at the lowest and two-to high concentrations of wort. The rest of the concentrations assayed did not show significant differences with respect to their concurrent controls.

Many factors affect longevity, among them are gender, genetic variation, environmental factors like diet, access to health care and traditions [77]. Fleming et al. [78] pointed out that Drosophila was a choice animal model to investigate longevity-promoting activities of a variety substances, like nutraceuticals, as flies seem to show many of the cellular senescence characteristics observed in mammals. Schriner et al. [79] showed that barley malts had the ability to increase the lifespan of Drosophila individuals, our results being in agreement for the lowest and highest concentrations tested showing an expansion of life of 7 and 8 days respectively. Unfortunately, no previous studies about lifespan have been found for the rest of the studied samples.

Regarding the healthspan results, all freeze-dried substances were able to improve the quality of life of flies at some concentrations tested, with respect to their concurrent control, except the highest concentration of Sladek hop, the lowest concentration of Saazer hop and *S. uvarum*, the second-to low concentration of wort and the second-to high concentration of Hopped wort that reduced the healthspan of *Drosophila* with respect to their concurrent controls. To the authors’ knowledge, this is the first time that healthspan has been studied for the tested compounds in the in vivo model organism, but there are previous studies with blond beer that showed beneficial effects in healthspan [42].

### 3.6. Cytotoxicity

**Figure 4:**
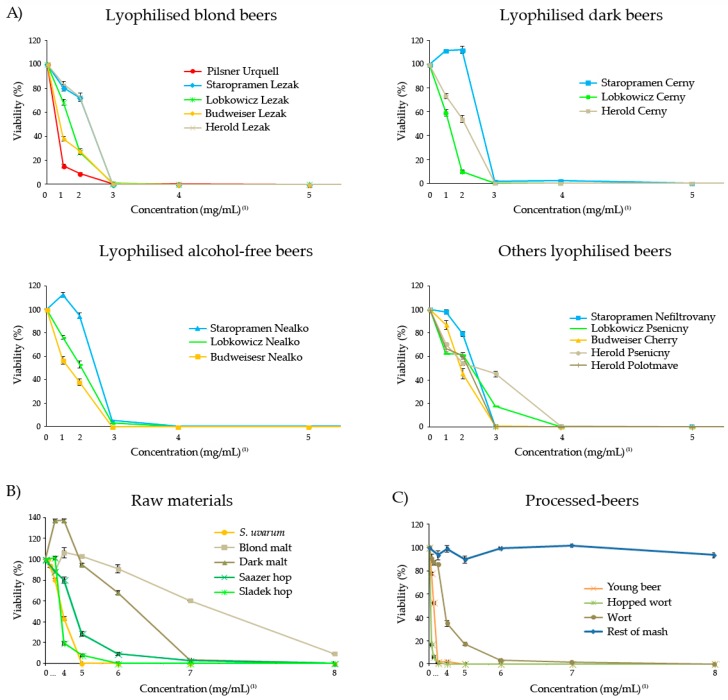
The effect of lyophilised Czech beers (**A**), raw materials (**B**) and processed-beers (**C**) on cell viability. The viability in the leukaemia cells (HL-60) treated with different concentrations of Czech lyophilised beers (**A**), raw materials (**B**) and processed-beers (**C**) for 72 h. Each point represents the growing percentage with respect to its control. The values indicate the mean from three independent experiments ± SE. ^(1)^ The value 1, 2, 3, 4, 5, 6, 7 and 8 correspond to the concentrations of 1.95, 3.9, 7.81, 15.625, 31.25, 62.5, 125 and 250 mg/mL for beers and their respective concentrations for each single component.

All substances assayed showed cytotoxic activity against the HL-60 cells except rest of mash which did not reach the inhibitory concentration of 50 (IC_50_) at any assayed concentration (Figure 4).

According to the IC_50_, lyophilised Czech beers are classified as follows: (i) Pilsner Urquell and Budweiser Lezak with a half maximal inhibitory concentration lower than 1.25 mg/mL; (ii) Lobkowicz Cerny followed by Budweiser Nealko, then Lobkowicz Lezak and finally Budweiser Cherry, with a IC_50_ between 1.25 and 3.5 mg/mL; (iii) Lobkowicz Nealko and Herold Cerny followed by Staropramen Cerny and Herold Polotmave, then Lobkowicz Nefiltrovany, followed by Herold Lezak, then Staropramen Nefiltrovany and finally Staropramen Nealko and Herold Psenicny with a IC_50_ ranging between 3.5 and 7.81 mg/mL. Despite this ranking, all beers were able to completely inhibit the tumour cells growth in a concentration that ranged between 7.81 and 15.625 mg/mL. The most cytotoxic raw material was Sladek hop followed by *S. uvarum*, Saazer hop, blond malt and dark malt reaching an IC_50_ at concentrations lower than the corresponding 15.625 mg/mL in beer.

Scientific studies indicate the cancer-prevention activity of beer. However, a variety of levels in the inhibition of tumour cell growth has been observed for different brands of beer, probably caused by the wide range in the components and in the antioxidant activities [53,80,81]. Nozawa et al. [82] showed that a moderate consumption of beer reduced the formation of colorectal tumours and preneoplastic lesions in azoxymethane to a significant level when experimental in vivo carcinogenesis was induced in male rats. Furthermore, the studies with a type of lyophilised blond lager beer showed a chemopreventive dose dependent response, reaching an IC_50_ at 125 mg/mL in the HL-60 cell line and an IC_50_ at 25 mg/mL in the immortal NIH3T3 cells [42].

Our results agree with the cytotoxic effects of beers, showing that Czech beers exhibit a stronger chemopreventive potential compared to other brands of beers as they reached an IC_50_ at lower concentrations than other beers. Moreover, although hops has been poorly studied by itself, some of its compounds like flavonols [83] and Xanthohumol [84] have shown anticarcinogenic effects.

### 3.7. DNA Fragmentation

**Figure 5:**
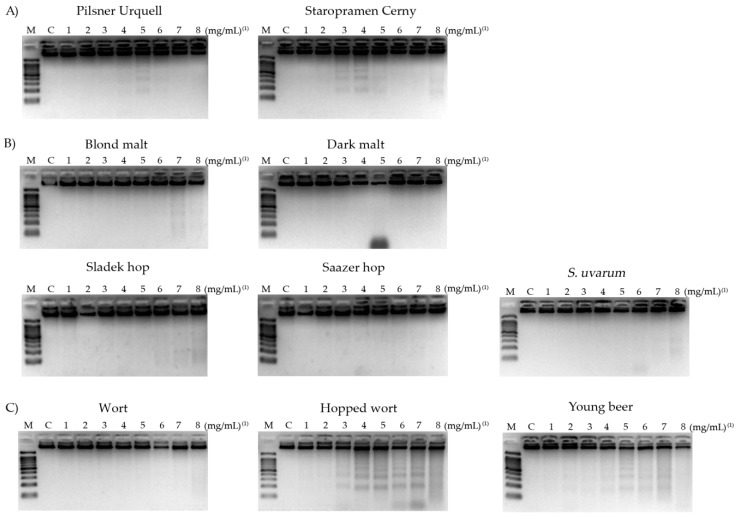
DNA damage induced by internucleosomal DNA fragmentation in promyelocytic HL-60 cells treated with different concentrations of a lyophilised Czech beer (**A**), raw materials (**B**) and processed-beers (**C**) for 5 h. M: DNA size marker. C: Control treatment. ^(1)^ The value 1, 2, 3, 4, 5, 6, 7 and 8 correspond to concentrations of 1.95, 3.9, 7.81, 15.625, 31.25, 62.5, 125 and 250 mg/mL for beers and their respective concentrations for each single component.

According to Wyllie, et al. [85], DNA internucleosomal breakage can be detected by a DNA band pattern related to the activation of the apoptotic way in cancer cells, being a hallmark of the genomic stability. The genomic integrity results of the HL-60 cells treated with lyophilised blond and stout Czech beers, raw materials and processed-beers are shown in Figure 5.

Pilsner Urquell showed a slight pattern of fragmentation when the cells were treated at concentrations of 15.625, 31.25 and 62.5 mg/mL (Figure 5A). A similar study with another brand of a lyophilised blond lager beer showed that beer induced DNA fragmentation damage at 15.625, 31.25 and 62.5 mg/mL concentrations [42]. The results obtained are similar to the results for Czech beers.

With respect to raw materials, all of them showed DNA damage at the concentration tested (Figure 5B), except blond malt and *S. uvarum* that induced a very light pattern of fragmentation at concentrations of 10 and 0.375 mg/mL (lane 7 and lane 8 in the agarose gel, respectively). Finally, processed-beer showed DNA fragmentation in a dose-dependent manner for hopped wort and young beer (Figure 5C).

Our study with the raw materials and processed-beers allow the suggestion of possible correlations between the modifications of beer and its compound properties during the brewing development. The most abundant phenols during the hopped wort process are xanthohumol and humulone, hop-phenols characterised by an induction of DNA damage as other studies have indicated [42,71]. During the beer maturing process, the characteristics of *Saccharomyces* and other phenols like ferulic acid, isoxanthohumol, vanillic acid, tyrosol, epicatechin gallate, among others, are involved in the final outcome of beers, reducing the proapoptotic DNA fragmentation activity as shown by matured beers [86]. In addition, some information about the prevention of the DNA damage capacity of hops could be found, but it was mainly due to one of its flavonoids [87,88], supporting in general our results of there being no induction of DNA-damage. Unfortunately, no prior information about malts on internucleosomal DNA fragmentation activity could be found.

### 3.8. Comet Assay

**Figure 6:**
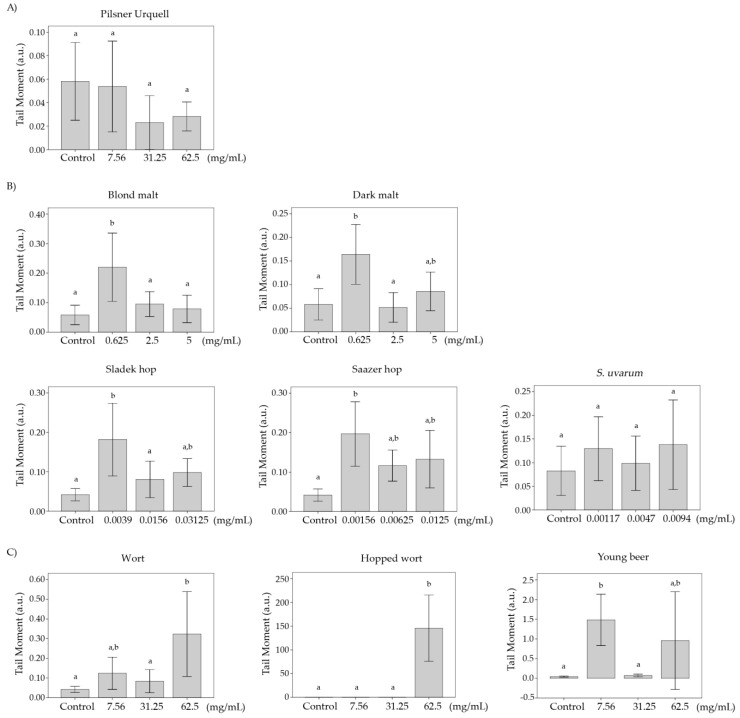
DNA strand breaks induction in promyelocytic HL-60 cells treated with different concentrations of a lyophilised Czech beer (**A**), raw materials (**B**) and processed-beers (**C**) for 5 h. Alkaline comet assay (pH > 13) of HL-60 cells treated. DNA migration is reported as the mean TM. The values are the mean ± SE. The different letters in the treatments mean the differences with respect to the negative control after a one-way ANOVA and post hoc Tukey’s test.

The averages and significances of the tail moment (TM) of HL-60 cells treated with blond and stout Czech lyophilised beers, raw materials and processed-beers are shown in Figure 6. The comet assay allows the ready identification of the apoptotic/necrotic cells based on changes in the nuclei morphology attributable to DNA damage at a unicellular level [89].

According to Fabiani et al. [90], the frequency distribution of DNA damage can be divided into five classes depending on the TM values as follows: Class 0, 1, 2, 3 and 4 with TM values <1; 1–5, 5–10, 10–20 and >20 corresponding to no damage, slightly damaged, medium damage, highly damaged and completely damaged DNA, respectively. Taking into account this classification, all concurrent control values fell into class 0 (TM < 1) showing no DNA damage in the untreated HL-60 cells. Pilsner Urquell, the raw materials and wort did not induce DNA damage at single and/or double strand breaks showing TM values lower than 1. On the other hand, young beer showed a slight damage at the lowest concentration assayed (7.56 mg/mL) with a TM value included in class 1. Moreover, Staropramen Cerny and hopped wort completely induced DNA damage at the medium and highest concentration respectively (31.25 and 62.5 mg/mL), with a TM value higher than 100 compared with their concurrent controls.

The results of DNA fragmentation fit with those obtained in the comet assay since the TM values higher than 30 mean that apoptosis mechanisms have been induced [91]. The highest concentration (62.5 mg/mL) of hopped wort tested showed a TM > 30 confirming the death of leukaemia cells by apoptosis as was observed in lane 5 and 6 of the fragmentation (Figure 5). The rest of compounds and concentrations tested showed a light or null DNA damage at double/single strands levels inducing cell death by a necrosis mechanism.

To the authors’ knowledge, no studies about the effects of beer and its components at an individual cellular level have been carried out. A study with a different brand of blond lager beer by Merinas-Amo et al. [42] showed significant damage with a TM higher than 100 when the HL-60 cells were treated with different concentrations of beer. However, this study did not obtain the same results for the lager beer. A similar clastogenic pattern was observed in the stout beer and hopped wort at the highest concentrations tested. Moreover, the different composition of beers allows the observation of a strong cytotoxic potential of full Czech beers compared with the studied blond lager beer, but not causing DNA damage at the cells during the 5 h of treatment and inducing total cell death at 72 h. It could be mainly because of the raw materials used (water, malt, hop and yeast), as those produced in the Czech Republic area are considered as some of the highest-quality materials in the world [6].

### 3.9. Methylation Status

**Figure 7:**
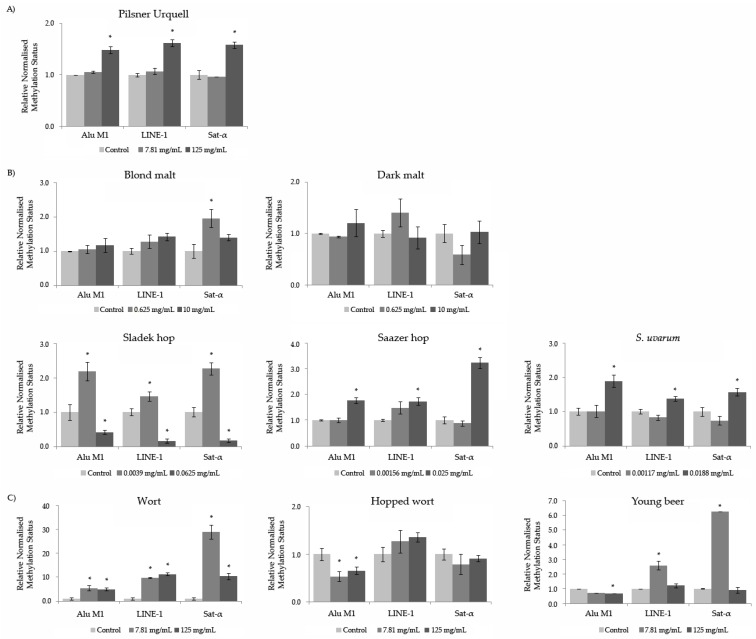
The relative normalised expression data of each repetitive element in treated HL-60 cells with different concentrations of a lyophilised Czech beer (**A**), raw materials (**B**) and processed-beers (**C**). The relative normalised expression data of each repetitive element (Alu M1, LINE-1 and Sat-α). The values represent the mean ± SE from three independent experiments. The untreated cells grown in RPMI were used as a control.

The results about the relative normalized expression of the Alu M1, LINE-1 and Sat-α repetitive sequences in the HL-60 cells treated with the compounds showed a general hypermethylation level has been observed, with some exceptions (Figure 7): Hopped wort showed a significant hypomethylating activity in the Alu M1 sequence at both studied concentrations, the highest concentration of Sladek hop and the highest concentration of Young beer treatments. Moreover, Dark malt exhibited no significant variation in the methylation level of the repetitive sequences studied. Contrarily, a significant hypermethylation was induced by all the Wort tested concentrations in all the genomic sequences studied: By Pilsner Urquell, Saazer hop and *S. uvarum* at the highest concentration of for all the sequences tested; by Sladek hop at lowest concentration for all the sequences studied; by Blond malt at lowest concentration assayed of Sat-α; and at the lowest concentration of Young beer for LINE and Sat-α repetitive sequences.

The control mechanisms of epigenetic DNA modifications are complex [92]. Endoparasitic repetitive sequences refrain from jumping around due to the repression of DNA methylation [93]. Most of the DNA methylation in humans occurs in the cytosine of CpG dinucleotides (CpG islands). In a normal cell, they are unmethylated, and the gene is expressed if the required transcription factors are present [93]. Due to the effects that methylation causes in the repetitive sequences of the genome, Pilsner Urquell, Saazer hop, *S. uvarum* and Wort at their highest concentration tested for all repetitive sequences studied, as well as lowest the concentration of Slaldek hop, Blond malt and Young beer, prevents DNA epigenetic damage, as methylation of the repetitive sequences is understood as a genomic protective mechanism [49,94]. These effects are not shown in the HL-60 cells when treated with Dark malt and at some concentrations and repetitive sequences of Sladek hop, Hopped wort and Young beer. Bearing in mind the idea of tumoral cell demethylation, it is suggested that beer and its compound act as preventive agents cutting off the repetitive sequences of tumour cells [94].

Unfortunately, there are no previous studies about the methylation status of lyophilised beers or raw materials. Merinas-Amo et al. [42] demonstrated that a lyophilised blond Lager beer induced hypermethylation in the DNA of tumour cells in a wide range of repetitive sequences. Fang et al. [95] suggested that the effect of a single polyphenol is not significant in normal dietary consumption, whereas the combination of polyphenols with dietary histone deacetylase inhibitors and the additive effect of different dietary chemicals may produce some effects. Furthermore, an excessive consumption of polyphenols in dietary supplements may affect the DNA methylation status [95]. The single or combined phenols contained in food should be taken into account in epigenetic-focussed therapies.

## 4. Conclusions

The new data corpus of our study contributes and supports the benefits showed by this lyophilized beverage, due to its safety, protection against an oxidative toxin, chemopreventive potential and the induction of DNA damage in tumour cells. A wide range of freeze-dried concentrations of beers and beer-processing substances have been tested and different biological activities have been observed in a multi-level set of in vivo and in vitro assays. Although several classes of volatile compounds like alcohol, ester, aldehydes, centone, etc. are lost in the lyophilisation [96], polyphenols and other bioactive components remain in the final sample [42]. All samples suffered the same lyophilisation process which makes it possible to properly compare the results among them in the different tests assayed.

Our work results indicate that raw materials and brewing conditions are key points for the final biological activities of beers. Despite the promising properties showed for all Czech beers, Pilsner Urquell, Budweiser Lezak, Lobkowicz Cerny and Budweiser Nealko were the beverages with the best results obtained in the in vivo and in vitro assays. The possible cause could be found in the absence of a Maillard reaction during the colouring of malt, which could induce a deterioration and an enhancement of food quality by the formation of antinutritional and toxic Maillard reaction products or beneficial compounds with antioxidant capacity.

Furthermore, the toxicological activities attributed to beer consumption cannot be related to a particular constituent because of their wide complexity and variability in their polyphenolic pattern, and also because the brewing is influenced by the raw materials, wort composition, yeast strains and fermentation conditions, among other factors.

On the whole, the positive properties in the in vivo (no toxic, no genotoxic, antitoxic, antigenotoxic, longevity and healthspan) and in the in vitro (cytotoxic and induction of DNA damage at different levels) tests are shown by the freeze-dried samples at different concentrations.

This study suggests that Czech beers as a drink have no severe potential adverse effects. However, further investigations are needed to clarify the effects of beer to other diets, as well as its important role in the prevention of chronic diseases, which mainly are related to the intake of antioxidants. Moreover, and despite the promising results obtained for the different freeze-dried beers and its materials, their consumption must be moderate due to the known negative effects induced by alcohol.

## Figures and Tables

**Table 1:** The samples of Czech beers and their ingredients used in the present research.

Beer Trade Mark	Named in the Manuscript	Beer Type/Ingredient Origin
Pilsner Urquell	Pilsner Urquell	Blond
Staropramen Lezak	Staropramen Lezak	Blond
Staropramen Cerny Lezak	Staropramen Cerny	Stout
Staropramen Nealko	Staropramen Nealko	Alcohol-free
Staropramen Nefiltrovany	Staropramen Nefiltrovany	Blond
Lobkowicz Lezak Premium	Lobkowicz Lezak	Blond
Lobkowicz Cerny Premium	Lobkowicz Cerny	Stout
Lobkowicz Nealkoholicky Premium	Lobkowicz Nealko	Alcohol-free
Lobkowicz Psenicny Premium	Lobkowicz Psenicny	Wheat
Budweiser Budvar B:Original, Svetly lezak	Budweiser Lezak	Blond
Budweiser Budvar B:Free, Nealkoholicke pivo	Budweiser Nealko	Alcohol-free
Budweiser Budvar B:Cherry, Tmavy lezak s visni	Budweiser Cherry	Cherry flavoured
Herold Svétly Breznicky Lezak	Herold Lezak	Blond
Herold Tmave Speciálni Pivo	Herold Cerny	Stout
Herold Psenicny Kvasnicovy Lezak	Herold Psenicny	Wheat
Herold Polotmave Specialni Pivo	Herold Polotmave	Pomegranate flavoured
Rest of mash ^(1)^	Rest of mash	CULS ^(8)^ Prague brewery
Blond malt	Blond malt	CULS Prague brewery
Dark malt	Dark malt	CULS Prague brewery
Sladek hop ^(2)^	Sladek hop	CULS Prague brewery
Saazer hop ^(3)^	Saazer hop	CULS Prague brewery
*Saccharomyces uvarum* ^(4)^	*S. uvarum*	CULS Prague brewery
Wort ^(5)^	Wort	CULS Prague brewery
Hopped wort ^(6)^	Hopped wort	CULS Prague brewery
Young beer ^(7)^	Young beer	CULS Prague brewery

^(1)^ Rest of malt after boiling process. ^(2)^ Hybrid aroma varieties of hop. ^(3)^ Fine aroma hop. ^(4)^ Lager yeasts sink to the bottom of the beer and ferment more slowly, preferring colder temperatures around 7–15 °C [29]. ^(5)^ Sugar solution obtained after filtration of barley malt boiling. ^(6)^ Liquid obtained after addition and boiling of hops to the wort, cooling and removal of spent hops. ^(7)^ Liquid obtained after yeast addition and fermentation during a week. ^(8)^ Czech University of Life Science.

**Table 2:** Primers information.

Reaction ID	GenBank Number	Amplicon Start	Amplicon End	Forward Primer Sequence 5′ to 3′ (N)	Reverse Primer Sequence 5′ to 3′ (N)	GC-Content (%)	
						Forward Reverse	
Alu C4	Consensus Sequence	1	98	GGTTAGGTATAGTGGTTTATATTTGTAATTTTAGTA (36)	ATTAACTAAACTAATCTTAAACTCCTAACCTCA (33)	25	27.3
Alu M1	Y07755	5059	5164	ATTATGTTAGTTAGGATGGTTTCGATTTT (29)	CAATCGACCGAACGCGA (17)	27.6	58.8
LINE-1	X52235	251	331	GGACGTATTTGGAAAATCGGG (21)	AATCTCGCGATACGCCGTT (19)	47.6	52.6
Sat-α	M38468	139	260	TGATGGAGTATTTTTAAAATATACGTTTTGTAGT (34)	AATTCTAAAAATATTCCTCTTCAATTACGTAAA (33)	23.5	21.2

Source: Weisenberger, Campan, Long, Kim, Woods, Fiala, Ehrlich and Laird [49].

**Table 3:** The genotoxicity of a lyophilised Czech beer, raw materials and processed-beers in the *Drosophila* wing spot test.

Compound	Clones per Wing (nº Spots) ^(1)^					
	N° of Wings	Small Single Clones (1–2 Cells) m = 2	Large Simple Clones (>2 Cells) m = 5	Twin Clones m = 5	Total Clones m = 2	*U*-Test ^(2)^
**H_2_O**	38	0.105 (4)	0.053 (2)	0	0.158 (6)	
**H_2_O_2_**	40	0.2 (8)	0.2 (8)	0	0.400 (16) +	
**Pilsner Urquell (mg/mL)**						
3.125	40	0.175 (7)	0.100 (4)	0	0.275 (11) i	∆
50	40	0.225 (9)	0.075 (3)	0	0.300 (12) i	∆
**Blond malt (mg/mL)**						
0.0625	40	0.225 (9)	0.025 (1)	0	0.250 (10) i	∆
1	40	0.250 (10)	0.075 (3)	0	0.325 (13) i	∆
**Dark malt (mg/mL)**						
0.0625	40	0.200 (8)	0.025 (1)	0	0.225 (9) i	∆
1	40	0.100 (4)	0.025 (1)	0	0.125 (5) i	∆
**Sladek hop (mg/mL)**						
0.00156	40	0.125 (5)	0.05 (2)	0	0.175 (7) i	∆
0.025	38	0.316 (12)	0.052 (2)	0	0.368 (14) i	∆
**Saazer hop (mg/mL)**						
0.000625	38	0.211 (8)	0.052 (2)	0	0.263 (10) i	∆
0.01	38	0.131 (5)	0.079 (3)	0	0.210 (8) i	∆
***S. uvarum* (mg/mL)**						
0.00023	40	0.200 (8)	0.075 (3)	0	0.275 (11) i	∆
0.00375	34	0.294 (10)	0.059 (2)	0	0.353 (12) i	∆
**Wort (mg/mL)**						
3.125	40	0.300 (12)	0	0.025 (1)	0.325 (13) i	∆
50	38	0.132 (5)	0.026 (1)	0	0.158 (6) i	∆
**Hopped wort (mg/mL)**						
3.125	40	0.200 (8)	0.025 (1)	0	0.225 (9) i	∆
50	38	0.131 (5)	0.158 (6)	0	0.289 (11) i	∆
**Young beer (mg/mL)**						
3.125	40	0.255 (9)	0	0	0.255 (9) i	∆
50	34	0.353 (12)	0	0	0.353 (12) i	∆

^(1)^ Statistical significances: + (positive); i (inconclusive) and + (positive) versus negative control. m multiplication factor. Kastenbaum-Bowman Test, error levels α = β = 0.05. ^(2)^ Inconclusive results were resolved by Mann Whitney *U*-test. Delta marker (∆) means no significant differences between treatment and concurrent control [35].

**Table 4:** Antigenotoxicity of a lyophilised Czech beer, raw materials and processed-beers in the *Drosophila* wing spot test.

	Clones per Wing (nº Spots) ^(1)^					Mann Whitney *U*-Test ^(2)^	Inhibition Percentage (%) ^(3)^
Compound	N° of Wings	Small Single Clones (1–2 Cells) m = 2	Large Simple Clones (More Than 2 Cells) m = 5	Twin Clones m = 5	Total Clones m = 2		
**H_2_O**	40	0.175 (7)	0	0	0.175 (7)		
**H_2_O_2_**	40	0.2 (8)	0.2 (8)	0	0.400 (16) +		
**Pilsner Urquell (mg/mL)**							
3.125	38	0.263 (10)	0.026 (1)	0	0.289 (11) β		27.75
50	40	0.200 (8)	0.025 (1)	0	0.225 (9) β		43.75
**Blond malt (mg/mL)**							
0.0625	40	0.225 (9)	0.050 (2)	0	0.275 (11) β		31.25
1	38	0.315 (12)	0.106 (4)	0	0.421 (16) λ	∆	−5.25
**Dark malt (mg/mL)**							
0.0625	40	0.175 (7)	0.05 (2)	0	0.225 (9) β		43.75
1	38	0.131 (5)	0.052 (2)	0	0.183 (7) β		54.25
**Sladek hop (mg/mL)**							
0.00156	38	0.131 (5)	0.079 (3)	0	0.210 (8) β		47.50
0.025	40	0.325 (13)	0.075 (3)	0	0.400 (16) λ	∆	0.00
**Saazer hop (mg/mL)**							
0.000625	38	0.079 (3)	0.052 (2)	0	0.131 (5) β		67.25
0.01	40	0.050 (2)	0.050 (2)	0	0.100 (4) β		75.00
***S. uvarum* (mg/mL)**							
0.00023	18	0.278 (5)	0.222 (4)	0	0.500 (9) λ	∆	−25.00
0.00375	27	0.222 (6)	0.125 (3)	0	0.333 (9) β		16.75
**Wort (mg/mL)**							
3.125	40	0.125 (5)	0.025 (1)	0	0.150 (6) β		62.50
50	40	0.250 (10)	0.025 (1)	0	0.275 (11) β		31.25
**Hopped wort (mg/mL)**							
3.125	40	0.325 (13)	0.075 (3)	0	0.400 (16) λ	∆	00.00
50	40	0.100 (4)	0.100 (4)	0	0.200 (8) β		50.00
**Young beer (mg/mL)**							
3.125	40	0.425 (17)	0.075 (3)	0	0.500 (20) λ	∆	−25.00
50	26	0.500 (13)	0.192 (5)	0	0.692 (18) λ	∆	−73.00

^(1)^ Statistical significance: + (positive) versus negative control; β (significantly different) and λ (inconclusive) versus positive control. m: multiplication factor. Kastenbaum-Bowman Test, error levels α = β = 0.05 [35]. ^(2)^ Inconclusive results were resolved by Mann Whitney U-test. Delta marker (∆) means no differences between the treatments and the concurrent control. ^(3)^ The inhibition percentage was calculated according to Abraham [39].

**Table 5:** The mean and significances of lifespan and healthspan.

Compound	Concentration	Mean Lifespan ^(1)^ (Days)		Mean Healthspan ^(1)^ (Days)	
Pilsner Urquell	Control	74.319		47.048	
	3.125 mg/mL	69.096	*	48.615	ns
	6.25 mg/mL	73.179	ns	45.385	ns
	12.5 mg/mL	74.571	ns	57.923	*
	25 mg/mL	64.857	*	44.765	ns
	50 mg/mL	72.413	ns	49.462	ns
Blond malt	Control	69.888		35.959	
	0.0625 mg/mL	76.349	*	47.769	*
	0.125 mg/mL	70.412	ns	39.714	ns
	0.25 mg/mL	78.448	ns	65.617	*
	0.5 mg/mL	73.514	ns	48.067	*
	1 mg/mL	77.946	*	53.583	*
Dark malt	Control	69.888		35.959	
	0.0625 mg/mL	70.557	ns	50.129	*
	0.125 mg/mL	66.314	*	34.135	ns
	0.25 mg/mL	58.281	*	35.692	ns
	0.5 mg/mL	73.077	ns	44.001	ns
	1 mg/mL	72.433	ns	38.176	ns
Sladek hop	Control	72.107		44.211	
	0.00156 mg/mL	77.439	*	50.792	ns
	0.00312 mg/mL	78.083	*	54.987	*
	0.00625 mg/mL	68.774	ns	45.750	ns
	0.0125 mg/mL	68.280	ns	42.154	ns
	0.025 mg/mL	67.556	ns	36.667	*
Saazer hop	Control	72.107		44.211	
	0.000625 mg/mL	60.714	*	29.929	*
	0.00125 mg/mL	69.993	ns	43.947	ns
	0.0025 mg/mL	70.314	ns	48.909	ns
	0.005 mg/mL	70.143	ns	44.754	ns
	0.01 mg/mL	72.098	ns	53.091	*
S. uvarum	Control	74.319		47.048	
	0.00023 mg/mL	67.590	*	42.545	*
	0.00047 mg/mL	73.738	ns	53.167	ns
	0.00094 mg/mL	71.876	ns	51.533	*
	0.00187 mg/mL	73.223	ns	55.292	*
	0.00375 mg/mL	79.060	*	54.393	*
Wort	Control	74.319		47.048	
	3.125 mg/mL	66.179	*	51.929	ns
	6.25 mg/mL	68.399	ns	38.143	*
	12.5 mg/mL	77.628	ns	64.238	*
	25 mg/mL	67.464	*	52.571	ns
	50 mg/mL	63.885	*	48.250	ns
Hopped wort	Control	74.319		47.048	
	3.125 mg/mL	72.232	ns	50.077	ns
	6.25 mg/mL	76.922	ns	63.375	*
	12.5 mg/mL	66.256	*	41.167	ns
	25 mg/mL	71.095	ns	40.541	*
	50 mg/mL	73.004	ns	53.833	ns
Young beer	Control	74.319		47.048	
	3.125 mg/mL	71.696	ns	47.357	ns
	6.25 mg/mL	70.747	*	57.357	*
	12.5 mg/mL	72.117	*	52.224	*
	25 mg/mL	78.426	ns	57.786	*
	50 mg/mL	75.314	ns	58.314	*

Means calculated by the Kaplan-Meier method and significances of the curves determined by the Log-Rank method (Mantel-cox). ^(1)^ ns: non-significant (*p* > 0.05), *: significant (*p* < 0.05) with respect to their concurrent controls.
